# The influence of antenatal betamethasone timing on neonatal outcome in late preterm infants: a single-center cohort study

**DOI:** 10.1007/s00404-024-07714-9

**Published:** 2024-09-09

**Authors:** Thomas Brückner, Anke Redlich

**Affiliations:** 1 Paediatrics, Medical Faculty, Otto-von-Guericke University, Leipziger Str. 44, 39120 Magdeburg, Sachsen-Anhalt Germany; 2 Present Address: Charité - Universitätsmedizin Berlin, SPZ-Neuropädiatrie, Augustenburger Platz 1, Campus: Ostring 1, 13353 Berlin, Germany; 3 Present Address: University Hospital for Obstetrics and Gynecology, Medical Faculty, Otto-von-Guericke University, Gerhart-Hauptmann Straße 35, 39108 Magdeburg, Sachsen-Anhalt Germany

**Keywords:** Betamethasone, Corticosteroids, Preterm birth, Respiratory distress syndrome, Newborn

## Abstract

**Purpose:**

Many pregnancies continue after antenatal corticosteroid exposure. Since long-term effects on late preterm neonatal outcome remain controversial, it remains unknown whether pregnant women who are at risk for preterm birth during the late preterm period and had prior antenatal corticosteroid exposure would benefit from an additional course of antenatal corticosteroids. We evaluated the need for future trials on this topic by comparing short term effects from antenatal betamethasone to long-term effects. We also examined the value of a risk-adapted approach.

**Methods:**

We observed neonatal outcomes in late preterm infants (34/0–36/0 weeks of gestation) who were exposed to antenatal betamethasone either up to 10 days prior birth (*n* = 8) or earlier in pregnancy (*n* = 89). We examined a real world population from the University Hospital Magdeburg (Germany) between 01 January 2012 and 31 December 2018, and a simulated high-risk population that was derived from the original data.

**Results:**

The indicators for relevant adverse outcomes did not differ in the unselected population. In the simulated high-risk population, recent antenatal corticosteroid administration significantly reduced the incidence of relevant cardiorespiratory morbidities (OR = 0.00, *p* = 0.008), and reduced the number needed to treat from 3.7 to 1.5.

**Conclusion:**

The superiority of recent antenatal corticosteroid administration in the late preterm period over earlier exposure strongly depended on the prevalence of respiratory disease. Before considering clinical trials on additional antenatal corticosteroid courses in the late preterm period, antenatal assessment tools to predict respiratory morbidity need to be developed.

**Supplementary Information:**

The online version contains supplementary material available at 10.1007/s00404-024-07714-9.

## What does this study add to the clinical work

**Table:** 

In an unselected population, we cannot support the use of additional betamethasone courses during the late preterm period. However, in a high-risk population that is not defined yet an additional betamethasone course may prevent severe respiratory disease.	

## Introduction

Antenatal corticosteroids (ACS) to prevent neonatal respiratory distress syndrome are standard of care for pregnant women at risk for immanent birth during the extreme (23/0–27/6 weeks of gestation), early (28/0–31/6) and moderate (32/0–33/6) preterm period. The use of ACS in the late preterm period (34/0–36/6 weeks) remains controversial. The upper limit to the appropriate gestational age was challenged by the Antenatal Late Preterm Steroid (ALPS) trial in 2016 [1], which reported significant effects on respiratory outcomes even after 34 weeks of gestation. The rate of late preterm and term infants exposed to ACS constantly increased in the aftermath of the ALPS trial [2, 3], although follow-up studies of randomized controlled trials (RCTs) reported increased risks for lower academic ability [4] and neurosensory deficits [5]. Ongoing concerns about mental and behavioral deficits result from large population studies, but are at serious risk for bias [6–9]. However, during the follow-up of the ALPS trial ACS was not associated with an adverse long-term developmental outcome [10]. Still, the current development towards a more liberate use of ACS raises the need for external evidence on safety and efficacy of ACS in different clinical scenarios. Besides pregestational diabetes pregnancies, major fetal malformations and twin pregnancies, the ALPS trial excluded women who received a prior course of ACS, to prevent contamination of the control group [1]. However, studies on the effect of prior ACS administrations on neonatal outcome in late preterm infants report conflicting results, ranging from dramatic improvement to worsening of respiratory morbidity [11–14]. None of these studies reported the time interval from ACS administration to birth.

For deliveries earlier than 34 weeks of gestation, ACS had an optimal effect on neonatal respiratory outcome when delivery occurred 2 to 7 days after administration [15–21]. However, the risk reduction for long-term sequelae seems to extend beyond that period [16–20], at least up to 10 days [22]. Thus the overall duration of protective ACS effects is difficult to determine. For ACS administration in the late preterm period there was no evidence for another increase of respiratory morbidity after 7 days [21]. Protective effects of prior ACS administrations may extend into the late preterm period, but prior ACS administrations may as well be an indicator for pregnancies, that are prone to adverse neonatal outcomes. In the clinical scenario of a pregnant woman at risk for preterm birth during the late preterm period, and prior ACS exposure, the risks and benefits from another ACS course remain unknown.

The objective of this study was to compare short-term with long-term effects of ACS on the neonatal outcome in a real-world late preterm population and in a simulated high-risk subpopulation. In this way, we wanted to determine the eligibility of future RCTs on additional ACS courses during the late preterm period in the context of prior ACS exposure. As a secondary objective we wanted to examine the value of a more individualized approach to ACS administrations in this context.

## Materials and methods

### Overview

We designed this study as an single-center cohort study at the University Hospital for Gynecology, Obstetrics and Reproductive Medicine in Magdeburg, Germany, in cooperation with the Neonatal Department.

We included all mother–child dyads of singleton pregnancies terminated at 34/0 to 36/6 weeks of gestation between 01 January 2012 and 31 December 2018 who received at least one course of intramuscular betamethasone anytime during pregnancy. Exclusion criteria were uncertainty of gestational age, major fetal malformations, other severe fetal inborn diseases and unpredictable corticosteroid effects. The full study protocol is available in the supplemental appendix.

We only used data previously generated in accordance with the patient’s written consent during hospital stay. The study was approved by the ethics committee of the Otto-von-Guericke University Magdeburg.

### Patient selection and cohort definition

We searched the birth registry for singleton pregnancies terminated at 34/0 to 36/6 weeks. We identified 526 mother and child dyads. All corresponding electronically archived medical records were manually reviewed by the first author. Inclusion and exclusion criteria were checked, cohort membership assigned, and data was recorded manually in a spreadsheet, according to the study protocol. We included 151 dyads who received betamethasone anytime during pregnancy, and assigned each dyad to one of two cohorts. The Recent Betamethasone (RB) cohort included all dyads that completed a course of betamethasone not more than 10 days prior to birth. The Past Betamethasone (PB) cohort included all dyads, in which the last betamethasone course was completed more than 10 days before delivery.

The decision to administrate betamethasone was made by the treating physicians, based on clinical judgement and the hospital’s Standard Operating Procedure (SOP) for antenatal betamethasone administration. We excluded nine mother-child dyads with neonates suffering from major malformations or severe inherited diseases, seven with uncertainties concerning the gestational age dating and one with an incomplete betamethasone trial at delivery. In principle 134 dyads were considered eligible for further evaluation. Eight dyads belonged to the RB cohort and 126 dyads belonged to the PB cohort.

Since the hospital’s SOP covered routine-ACS administrations only until the age of 33/6 weeks, the RB cohort was more likely to be of younger gestational age. This was confirmed by our first comparison of pregnancy characteristics. We restricted the PB cohort to the maximum gestational age of the RB cohort to eliminate this important confounder. A total of 97 dyads with gestational ages ranging from 34/0 to 36/0 weeks were ultimately included in the final analysis. All dyads had completed at least one course (2 doses) of intramuscular betamethasone 12 mg, either 12 h (fast track) or 24 h apart. Four dyads in the PB cohort had completed a second course of betamethasone at 25/0, 25/1, 25/6 and 27/3 weeks, respectively. No dyad had more than 2 courses of antenatal betamethasone.

To simulate a high-risk population we selected those dyads with any need for respiratory support in the neonate from the original dataset.

### Data abstraction

Personal data was pseudonymized during data recording and analysis, and anonymized for long-term storage. We defined 43 items to describe pregnancy characteristics and the maternal risk profiles, and 92 items to characterize the neonatal population and their outcomes. Details about item definition are described in the study protocol. Modes of delivery were grouped into cesarean section and other. However, all neonates were either subject to spontaneous vaginal delivery or cesarean section. The only neonate born by vacuum extraction dropped out during the restriction of the PB cohort to the maximum gestational age of 36/0 weeks. There was no neonate born by forceps. The neonatal outcome was divided into 10 different categories: neonatal outcome—biometric, death, cardiorespiratory, infection, neurologic, metabolic, icterus, feeding, temperature regulation and hospital stay. All items were manually recorded from electronically archived medical records by the first author. Unavailable item data were left empty, and the affected cases were excluded during the analysis of the individual item.

We refrained from assigning the disease labels “wet lung” or “respiratory distress syndrome”, because unambiguous definition criteria were difficult to meet. In this regard, the absence of chest X-rays in most cases was the main limiting factor. Instead, we focused on the duration of respiratory support and the use of surfactant as surrogate, but more valid parameters.

### Composite outcomes

For all categories except Biometric, Infection and Icterus a composite outcome was generated to indicate any adverse events of special interest. Composite outcome-death was defined as stillbirth or neonatal death up to 28 days of life. Composite outcome-cardiorespiratory was defined as any of the following parameters: continuous positive airway pressure (CPAP) or high flow nasal cannula (HFNC) >2 h, need for inspired oxygen fraction (FiO_2_) >0.3 >4 h, mechanical ventilation, external membrane oxygenation (ECMO), surfactant application, pneumothorax, bronchopulmonal dysplasia (BPD) or arterial hypotension. Composite outcome-neurologic was defined as the incidence of any intracranial bleeding, encephalopathy or neonatal seizures. Composite outcome-metabolic was defined as either severe hypoglycemia or need for glucose containing i.v. solutions. Composite outcome-feeding was defined as either ablactation or need for additional formula feds at dismission/transfer to another hospital. Composite outcome-temperature regulation was defined as either need for external heat or the lack of rooming-in on the first day of life. Composite outcome-hospital stay was defined as Neonatal Intensive Care Unit (NICU) stay of any duration, hospital stay >21 days or transfer to another hospital (lost to follow-up). The composite outcome-combined was defined as the occurrence of any composite outcome. Composite outcome-BIAS was defined as any of the following: transfer to another hospital, discharge against physician’s advice or incomplete documentation.

### Data analysis

For categorial data, we display the number of affected cases in total numbers and in percent. Differences between both cohorts were tested for significance with Fisher’s test. The effect size is described by the odds ratio. For all numeric variables we assumed a nonparametric model. For numeric data, we display median and range. To determine the effect size we calculated the difference in arithmetic means. Differences in numeric parameters between the two cohorts were tested for significance using the Brunner–Munzel test. A type I error <0.05 was considered to be significant.

All analyses were performed using the “R” software environment version 3.6.3 (Linux Kernel 5.4.0-139-generic on an Intel Core i7 CPU).

## Results

### Pregnancy characteristics and risk profiles

Mothers in the PB cohort had a higher rate of abortions prior to the current pregnancy. We found no other differences in pregnancy characteristics (Tables 1, 2). In the RB cohort there were non-significant trends towards more deliveries by cesarean section, more deliveries for medical indication, Streptococcus B colonization, pregnancies with intrauterine growth retardation and higher rates of rhesus incompatibility with positive Coombs test.

**Table 1:** Corrected maternal and pregnancy characteristics

	Recent betamethasone		Past betamethasone		$\Delta$mean	*p*-value
	Median	(Range) [*n* = 8]	Median	(Range) [*n* = 89]		
Maternal age [a]	28.5	(22–36)	30	(17–41)	– 0.93	0.558
Body mass index [kg/m^2^]	25.7	(15.1–29.4)	23.1	(17.9–42.3)	– 0.86	0.798
Gestational age [d]	240.5	(239–252)	244	(238–252)	– 1.98	0.303
Gravida [*n*]	2	(1–4)	2	(1–7)	0.00	0.652
Para [*n*]	1	(1–3)	1	(1–5)	– 0.17	0.550
Miscarriages [*n*]	1	(0–1)	0	(0–2)	0.37	0.077
Abortions [*n*]	0	(0–0)	0	(0–3)	– 0.17	<0.001
Stillbirths [*n*]	0	(0–0)	0	(0–1)	– 0.01	0.320
Cesarean sections [*n*]	1	(0–3)	1	(0–4)	0.12	0.675
Latency to bethamethasone [d]	7.5	(0–10)	34	(11–80)	– 33.73	<0.001
GA at betamethasone [d]	235.5	(230–252)	209	(170–236)	31.39	<0.001

**Table 2:** Pregnancy risk profiles

	Recent betamethasone		Past betamethasone		Odds ratio	*p*-value
	No.	(%) [*n* = 8]	No.	(%) [*n* = 89]		
Cesarean section	7	(87.5)	56	(62.9)	4.08	0.254
Assisted reproduction	0	(0.0)	7	(7.9)	0.00	1.000
Smoking in pregnancy	2	(25.0)	19	(21.3)	1.23	1.000
Prior pregnancy complications	1	(12.5)	26	(29.2)	0.35	0.437
Prior birth complications	1	(12.5)	22	(24.7)	0.44	0.676
Pregnancy at risk	8	(100.0)	86	(96.6)	Inf	1.000
Medically indicated delivery	7	(87.5)	43	(48.3)	7.36	0.060
Gestational diabetes	0	(0.0)	15	(16.9)	0.00	0.351
Insulin therapy	0	(0.0)	7	(7.9)	0.00	1.000
Makrosomia	0	(0.0)	1	(1.1)	0.00	1.000
Birth arrest/mismatch	0	(0.0)	10	(11.2)	0.00	1.000
Gestational hypertonus	1	(12.5)	6	(6.7)	1.96	0.464
Preeclampsia	0	(0.0)	7	(7.9)	0.00	1.000
HELLP-syndrome	1	(12.5)	4	(4.5)	2.98	0.356
Gestational hepatosis	0	(0.0)	0	(0.0)	0.00	1.000
Intrauterine growth retardation	3	(37.5)	18	(20.2)	2.34	0.365
Prior uterine operation	1	(12.5)	26	(29.2)	0.35	0.437
Uterine malformation	0	(0.0)	5	(5.6)	0.00	1.000
Chorionic villus sampling	0	(0.0)	1	(1.1)	0.00	1.000
Bleeding in pregnancy	0	(0.0)	25	(28.1)	0.00	0.108
Strapping	0	(0.0)	3	(3.4)	0.00	1.000
Anemia during pregnancy	5	(62.5)	50	(56.2)	1.30	1.000
Rhesus incompatibility	1	(12.5)	6	(6.7)	1.96	0.464
Coombs test positive	1	(33.3)	1	(12.5)	3.06	0.491
Amiotic infection syndrome	0	(0.0)	1	(1.1)	0.00	1.000
Suspected triple I	0	(0.0)	0	(0.0)	0.00	1.000
Confirmed triple I	0	(0.0)	0	(0.0)	0.00	1.000
STORCHL-infection	0	(0.0)	2	(2.2)	0.00	1.000
Streptococcus B colonization	1	(50.0)	6	(12.0)	6.87	0.253
Bleeding immediately prior to birth	0	(0.0)	1	(1.1)	0.00	1.000
Partial premature placental abruption	0	(0.0)	4	(4.5)	0.00	1.000
Premature placental abruption	0	(0.0)	2	(2.2)	0.00	1.000

### Characteristics of the neonatal population

The lower head circumferences in the RB cohort reached significance, but not the dedicated z scores. There were non-significant trends towards lower birthweight and length, as well as more Small-for-Gestational-age children in the RB cohort (Table 3).

**Table 3:** Neonatal characteristics

	Recent betamethasone		Past betamethasone		$\Delta$Mean	*p*-value
	Median	(Range) [*n* = 8]	Median	(Range) [*n* = 89]		
Birthweight [g]	2020	(1200–2720)	2320	(1390–3420)	– 307.32	0.151
Birthweight z-score	– 0.82	(– 2.76 to 0.42)	– 0.46	(– 2.47 to 1.33)	– 0.52	0.422
Length at birth [cm]	44	(29.5–50)	46.5	(38–51)	– 2.89	0.257
Length at birth z-score	– 0.83	(– 5.9 to 0.96)	– 0.45	(– 3.39 to 1.32)	– 0.87	0.464
Head circumference [cm]	30.25	(26.5–33)	32	(28–36)	– 1.61	0.047
Head circumference z-score	– 1.46	(– 3.46 to 0.24)	– 0.56	(– 3.35 to 2.17)	– 0.86	0.169

	No.	(%) [*n* = 8]	No.	(%) [*n* = 89]	Odds ratio	*p*-value
Assigned sex (m:f)	4:4	(50:50)	48:41	(54:46)	0.86	1.000
Small-for-gestational-age	3	(37.5)	19	(21.3)	2.19	0.376

### Neonatal outcome-cardiorespiratory

**Fig. 1:**
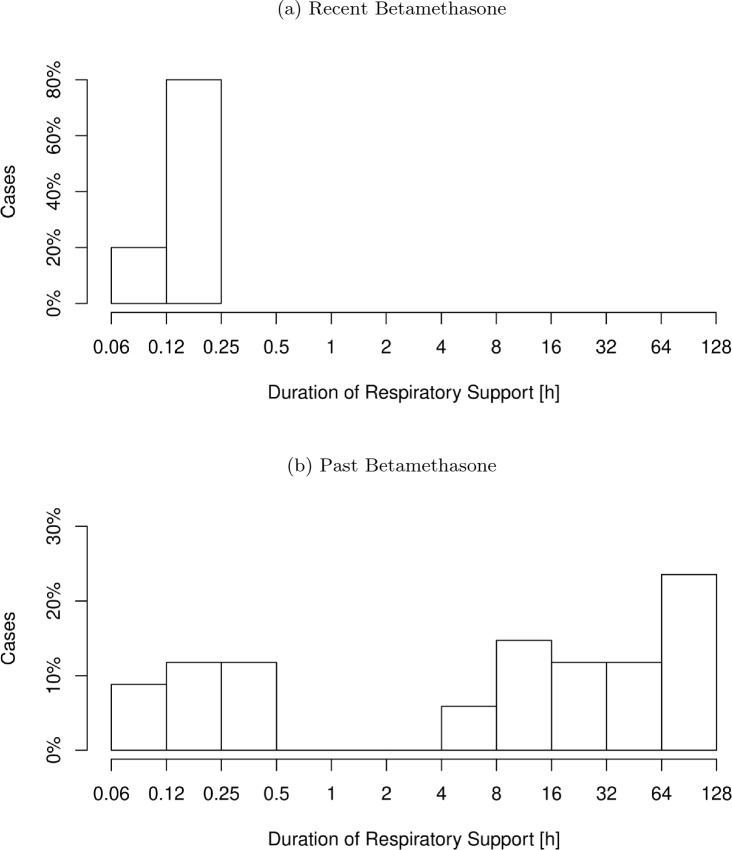
**a**) Distribution of respiratory support duration, when betamethasone was administered up to 10 days prior birth. **b**) Distribution of respiratory support duration, when betamethasone was administered more than 10 days prior birth. The second peak of respiratory support duration > 4 h vanished, when betamethasone was administered up to 10 days prior birth

The RB cohort had significantly less days on caffeine treatment, but with a barely relevant effect size. Non-significant trends showed an increased number of neonates with respiratory distress during the first 2 h of life in the RB cohort (62.5% vs. 44.9%) with a reduction of mean respiratory support duration in the RB cohort by about 11 h at the same time. Not a single case in the RB cohort needed respiratory support longer than 2 h in contrast to 24.7% in the PB cohort. A relevant adverse cardiorespiratory outcome, as indicated by the Cardiorespiratory Composite Outcome, occured in 27% in the PB cohort, while there was not a single case in the RB cohort (Table 4). The distribution of respiratory support duration shows two clusters in the PB cohort, probably referring to different etiologic entities. In the RB cohort the cluster with longer respiratory support vanished (Fig. 1).

In the simulated high-risk population there was a significant reduction of respiratory support duration by 28.8 h (*p*< 0.001). Need for CPAP/HFNC >2 h did not occur in the RB cohort subpopulation compared to 64.7% in the PB cohort (*p* = 0.011). This results in a decrease of the Cardiorespiratory Composite Outcome from 67.6% to 0.0% (*p* = 0.008), compared to 27.0% to 0.0% (*p* = 0.194) in the general late preterm population (Table 5). This translates into a reduction of the Number Needed to Treat (NNT) from 3.7 to 1.5, when ACS administration would be provided only for infants with postnatal need for respiratory support.

**Table 4:** Neonatal outcome-cardiorespiratory

	Recent betamethasone		Past betamethasone		Odds ratio	*p*-value
	No.	(%) [*n* = 8]	No.	(%) [*n* = 89]		
Supported transition	4	(50)	27	(30.3)	2.27	0.263
Respiratory transition disorder	5	(62.5)	40	(44.9)	2.03	0.466
Intubation	0	(0)	2	(2.2)	0.00	1.000
HighFlow nasal cannula	0	(0)	3	(3.4)	0.00	1.000
Continuous positive airway pressure	5	(62.5)	33	(37.1)	2.80	0.256
Inspired oxygen fraction> 0.3	1	(12.5)	18	(20.2)	0.57	1.000
Any respiratory support	5	(62.5)	34	(38.2)	2.67	0.261
CPAP/HFNC > 2 h	0	(0)	22	(24.7)	0.00	0.192
CPAP/HFNC > 12 h	0	(0)	16	(18)	0.00	0.346
FiO_2_ > 0.3 longer than 4 h	0	(0)	3	(3.4)	0.00	1.000
FiO_2_ > 0.3 longer than 24 h	0	(0)	0	(0)	0.00	1.000
Extracorporal membrane oxygenation	0	(0)	0	(0)	0.00	1.000
Surfactant	0	(0)	1	(1.1)	0.00	1.000
Apnoe	2	(25)	19	(21.3)	1.23	1.000
Pneumothorax	0	(0)	0	(0)	0.00	1.000
Mekonium aspiration syndrome	0	(0)	0	(0)	0.00	1.000
Bronchopulmonary dysplasia	0	(0)	0	(0)	0.00	1.000
PPHN	0	(0)	0	(0)	0.00	1.000
Persistent fetal circulation	0	(0)	0	(0)	0.00	1.000
Arterial hypotension	0	(0)	4	(4.5)	0.00	1.000
Hemodynamically relevant PDA	0	(0)	0	(0)	0.00	1.000
PDA: ibuprofene therapy	0	(0)	0	(0)	0.00	1.000
Composite outcome: cardiorespiratory	0	(0)	24	(27)	0.00	0.194

	Median	(Range) [*n* = 8]	No.	(Range) [*n* = 89]	$\Delta$Mean	*p*-value
Umbilical artery pH	7.34	(7.23–7.37)	7.32	(7.16–7.46)	0.00	0.898
Umbilical vein pH	7.38	(7.29–7.40)	7.38	(7.21–7.55)	– 0.01	0.908
APGAR min. 1	8	(5–9)	9	(4–10)	– 0.72	0.140
APGAR min. 5	9	(7–10)	10	(6–10)	– 0.23	0.503
APGAR min. 10	10	(9–10)	10	(7–10)	0.21	0.451
Invasive ventilation [h]	0	(0–0)	0	(0–92)	– 1.07	0.158
HFNC [h]	0	(0–0)	0	(0–51)	– 0.82	0.083
CPAP [h]	0.11	(0–0.2)	0	(0–95.5)	– 9.08	0.613
Overall need for respiratory support [h]	0.11	(0–0.2)	0	(0–109.25)	– 10.97	0.693
Inspired oxygen fraction >0.3 [h]	0	(0–0.02)	0	(0–22.75)	– 0.58	0.375
Treatment with caffeine [d]	0	(0–0)	0	(0–15)	– 0.98	< 0.001
Need for volume administration [*n*]	0	(0–0)	0	(0–1)	– 0.03	0.083
Need for catecholamine treatment [d]	0	(0–0)	0	(0–4)	– 0.04	0.320

**Table 5:** Subgroup analysis–neonates with adverse respiratory outcome

	Recent betamethasone		Past betamethasone		Odds ratio	*p*-value
	No.	(%) [*n* = 5]	No.	(%) [*n* = 34]		
Supported transition	4	(80)	27	(79.4)	1.04	1.000
Respiratory transition disorder	5	(100)	32	(94.1)	Inf	1.000
Intubation	0	(0)	2	(5.9)	0.00	1.000
HighFlow nasal cannula	0	(0)	3	(8.8)	0.00	1.000
Continuous positive airway pressure	5	(100)	33	(97.1)	Inf	1.000
Inspired oxygen fraction > 0.3	1	(20)	18	(52.9)	0.23	0.342
Any respiratory support	5	(100)	34	(100)	0.00	1.000
CPAP/HFNC > 2 h	0	(0)	22	(64.7)	0.00	0.011
CPAP/HFNC > 12 h	0	(0)	16	(47.1)	0.00	0.066
FiO_2_ > 0.3 longer than 4 h	0	(0)	3	(8.8)	0.00	1.000
FiO_2_ > 0.3 longer than 24 h	0	(0)	0	(0)	0.00	1.000
Extracorporal membrane oxygenation	0	(0)	0	(0)	0.00	1.000
Surfactant	0	(0)	1	(2.9)	0.00	1.000
Apnoe	2	(40)	14	(41.2)	0.95	1.000
Pneumothorax	0	(0)	0	(0)	0.00	1.000
Mekonium aspiration syndrome	0	(0)	0	(0)	0.00	1.000
Bronchopulmonary dysplasia	0	(0)	0	(0)	0.00	1.000
PPHN	0	(0)	0	(0)	0.00	1.000
Persistent fetal circulation	0	(0)	0	(0)	0.00	1.000
Arterial hypotension	0	(0)	3	(8.8)	0.00	1.000
Hemodynamically relevant PDA	0	(0)	0	(0)	0.00	1.000
PDA: ibuprofene therapy	0	(0)	0	(0)	0.00	1.000
Composite outcome: cardiorespiratory	0	(0)	23	(67.6)	0.00	0.008

	Median	(Range) [*n* = 5]	Median	(Range) [*n* = 34]	$\Delta$Mean	*p*-value
Umbilical artery pH	7.34	(7.23–7.37)	7.32	(7.22–7.46)	– 0.01	0.895
Umbilical vein pH	7.36	(7.29–7.40)	7.38	(7.27–7.55)	– 0.03	0.372
APGAR min. 1	7	(5–8)	7	(4–10)	– 0.55	0.429
APGAR min. 5	8	(7–9)	8	(6–10)	0.11	0.789
APGAR min. 10	10	(9–10)	9	(7–10)	0.66	0.057
Invasive ventilation [h]	0	(0–0)	0	(0–92)	– 2.80	0.160
HFNC [h]	0	(0–0)	0	(0–51)	– 2.14	0.083
CPAP [h]	0.17	(0.08–0.2)	10.54	(0–95.5)	– 23.86	< 0.001
Overall need for respiratory support [h]	0.17	(0.08–0.2)	12.38	(0.07–109.25)	– 28.80	< 0.001
Inspired oxygen fraction > 0.3 [h]	0	(0–0.02)	0.05	(0–22.75)	– 1.53	0.007
Treatment with caffeine [d]	0	(0–0)	0	(0–15)	– 1.85	< 0.001
Need for volume administration [*n*]	0	(0–0)	0	(0–1)	– 0.06	0.160
Need for katecholamine treatment [d]	0	(0–0)	0	(0 - 4)	– 0.12	0.325

### Neonatal outcome-metabolic

The analysis for hypoglycemia related problems showed significantly longer administration of glucose containing intravenous solutions in the RB cohort. There was a non-significant trend towards more hypoglycemia, severe hypoglycemia and need for glucose substitution. No neurologic sequelae of hypoglycemia was reported during the neonatal period.

### Neonatal outcome-icterus

The RB cohort received significantly less photo therapy. Lower maximum bilirubin levels in the RB cohort closely missed significance.

### Neonatal outcome-feeding

There were no significant differences in the feeding category. Non-significant trends suggested prolonged gastric tube feeding and gastroesophageal reflux in the RB cohort.

### Neonatal outcome-temperature regulation

The need for incubator stay was evenly distributed between both cohorts. On average, neonates in the RB cohort stayed 2.81 days longer in warm beds/under heat lamps, closely failing significance.

### Neonatal outcome-hospital stay

A significant difference between both cohorts was detected for weight at discharge. On average, neonates in the RB cohort were 271.2 g lighter than the PB cohort at similar gestational ages. All other items did not reach significance. Not a single neonate in the RB cohort was eligible for rooming-in on the first day of life in contrast to 25.8% of neonates in the PB cohort. The need for a neonatal intensive care unit (NICU) stay was 50% in the RB cohort vs. 30.3% in the PB cohort. The mean duration of NICU stay was similar between both cohorts.

### Neonatal outcome-other

There were no stillbirths or deaths during the neonatal period in either cohort. There was no significant difference in the rate of infections or infection related diseases. No serious neurological events were reported.

### Neonatal outcome—composite outcomes

Composite Outcome parameters (see Composite Outcomes) did not show any significant differences in the original population (Table 6). The cardiorespiratory composite outcome was significantly in favor of the RB cohort in the simulated high-risk cohort as mentioned above (see Neonatal outcome—composite outcomes).

**Table 6:** Neonatal outcome—composite outcomes

	Recent betamethasone		Past betamethasone		Odds ratio	*p*-value
	No.	(%) [*n* = 8]	No.	(%) [*n* = 89]		
Composite outcome: death	0	(0.0)	0	(0.0)	0.00	1.000
Composite outcome: cardiorespiratory	0	(0.0)	24	(27.0)	0.00	0.194
Composite outcome: neurologic	0	(0.0)	2	(2.2)	0.00	1.000
Composite outcome: metabolic	6	(75.0)	45	(50.6)	2.90	0.274
Composite outcome: feeding	5	(62.5)	55	(61.8)	1.03	1.000
Composite outcome: temperature regulation	8	(100.0)	66	(74.2)	Inf	0.192
Composite outcome: hospital stay	4	(50.0)	33	(37.1)	1.69	0.476
Composite outcome: combined	8	(100.0)	79	(88.8)	Inf	1.000

## Comment

### Principal findings

The composite outcome parameters, indicating relevant adverse events, did not show any significant differences between both cohorts.

In our simulated high-risk population recent ACS administration was associated with a significant and relevant reduction of severe respiratory disease, and a reduced Number Needed to Treat. However, we are not yet able to reliably identify this subpopulation in the antenatal setting.

### Context

Known risk-factors for adverse neonatal outcome in late preterm infants are lower gestational age [23–25], low birthweight [24, 26–28], male fetus [27, 29], gestational diabetes and large-for-gestational-age [24, 30, 31], chorioamnionitis [31], medically indicated delivery [32] and cesarean section [24, 33–35]. As reported in Section "Pregnancy Characteristics and Risk Profiles" we did not find any significant group differences, concerning these possible confounders. Considering non-significant trends, the RB cohort was affected by a higher prevalence of these risk-factors, except for gestational diabetes. Given the lower rate of Composite Cardiorespiratory Outcome in the RB cohort, we do not believe in a relevant confounding influence. We were not able to show a significant correlation between recent ACS administration and hypoglycemia, but the prolonged need for glucose containing intravenous solutions in the RB cohort may indicate an impaired glucose metabolism. Gulersen et al. demonstrated that the risk for hypoglycemia is highest in the group of late preterm infants delivered timely after ACS administration [21]. Many of the trends reported, including hypoglycemia, may be confounded by low birthweights. Low birthweights were reported to occur in the context of ACS [36, 37] and even the most recent reviews still include trends towards lower birthweights similar to our findings [38, 39]. We were not able to determine whether low birth biometrics resulted from recent ACS administration or were a preexisting condition. As low birthweight may not be independent of ACS, a corrected analysis was omitted.

Despite higher rates of rhesus incompatibility and positive Coombs tests, the maximum bilirubin levels were significantly lower in the RB cohort, and those neonates needed less phototherapy. Positive effects of ACS on the need for phototherapy were shown previously by Porto et al. [40].

We found no adverse neurologic effects of hypoglycemia in the neonatal period, but we cannot tell about possible long-term effects.

### Clinical and research implications

We cannot support unselected rescue-ACS administrations in the late preterm period on the current data basis. Positive effects on the respiratory morbidity in late preterm infants strongly depend on the prevalence of respiratory disease. Therefore, randomized controlled trials on rescue ACS administrations in the late preterm period should focus on high-risk populations. We suggest to put more efforts into the development of clinical prediction tools to determine the risk of respiratory disease late preterm infants.

### Strengths and limitations

One strength of our study is the comparability of both cohorts. After correction for gestational age, both cohorts were considered to be comparable. The higher rate of abortions prior to the current pregnancy in the PB cohort was judged to be irrelevant to the neonatal outcome.The generalizability of our results benefits from the real world population with low dropout rates, but is limited by the single-center approach. The main limitation of our study is the low number of cases in the RB cohort, because ACS administrations were handled very strict at our center. Therefore real significances may easily be missed. On the other hand we wanted to display a wide range of outcome parameters of possible interest, that may interact with each other in different ways. This makes our study prone to the risk of false-significant results due to alpha-error accumulation. The condensation of single parameters into composite outcome parameters, indicating relevant adverse outcomes, somewhat attenuates the impact of alpha-error accumulation, if we strictly focus on the composite outcome parameters in the final interpretation. As our objective was to give guidance to future study design, we prioritized the identification of relevant outcome parameters over the avoidance of alpha-error accumulation. Another limitation is the retrospective study design, that limited data quality and availability. We were unable to obtain data about the individual indication for ACS administration and estimated birthweights, and we had only limited access to Body Mass Indices (BMI) in the early pregnancy (44.3% of all cases with missing data).

### Conclusion

A risk-adapted approach to additional ACS-administrations in the late preterm period may yield the potential to address adverse long-term neurodevelopmental effects resulting from neonatal respiratory disease, while lowering the number of ACS exposures. Before considering clinical trials on additional antenatal corticosteroid courses in the late preterm period, antenatal assessment tools to predict respiratory morbidity need to be developed.

## Supplementary Information

Below is the link to the electronic supplementary material.

Supplementary file 1 (pdf 0 KB)

Supplementary file 2 (pdf 0 KB)

## Data Availability

The original dataset and R code are available at 10.17632/yg5cyxfrjy.1.

## Declarations

### Use of AI technology

We implemented some suggestions of the AI-tool "Curie" hosted by AJE^®^ to improve language quality and style.
