# Supplementary material for: The influence of antenatal betamethasone timing on neonatal outcome in late preterm infants: a single-center cohort study
Source: Arch Gynecol Obstet. 2024 Sep 9;311(4):1017–27. doi: 10.1007/s00404-024-07714-9 (PMC11985642; doi:10.1007/s00404-024-07714-9)
Supplement: Supplementary file 1 — (pdf 0 KB) [file 404_2024_7714_MOESM1_ESM.pdf]

Archives of Gynecology and Obstetrics: The Influence of Antenatal Betamethasone Timing on Neonatal Outcome in Late Preterm Infants Supplemental Information: Study Protocol

Thomas Brückner[1*] and Anke Redlich[2]

[1*]Paediatrics, Medical Faculty, Otto-von-Guericke University, Leipziger Str. 44, Magdeburg, 39120, Sachsen-Anhalt, Germany.
[2]University Hospital for Obstetrics and Gynecology, Medical Faculty, Otto-von-Guericke University, Gerhart-Hauptmann Straße 35, Magdeburg, 39108, Sachsen-Anhalt, Germany.

*Corresponding author(s). E-mail(s): thomas.brueckner@charite.de;
Contributing authors: anke.redlich@med.ovgu.de;

*Present Address:
Charité - Universitätsmedizin Berlin, SPZ-Neuropädiatrie, Augustenburger Platz 1, Campus: Ostring 1, 13353 Berlin, Germany.

The Influence of Antenatal Betamethasone Timing on the Neonatal Outcome in Late Preterm Infants
# – Study Protocol –

T. Brückner, A. Redlich

June 2023

Contents

1 Background **4**

2 Goal **5**

3 Question **5**

4 Design **6**

4.1 Overview 6

4.2 Inclusion Criteria 6

4.3 Exclusion Criteria 6

4.4 Cohort Assignment 7

5 Item Selection **8**

5.1 Maternal Data 8

5.2 Infant Data 10

5.3 Risk for BIAS 15

5.4 Composite Outcomes 16

6 Analysis **17**

7 Funding and Conflicting Interests **18**

1 Background

Scince Liggins et al conducted the first human trial on antenatal corticosteroids (ACS) for fetal lung maturation in 1972[1], their use in pregnancies at risk for preterm delivery became standard up to 34 weeks of gestation, as recommended by several clinical practice guidelines[2–8].

Safety and efficacy of ACS in pregnancies at risk for preterm birth is supported by several cochrane reviews. However, evidence from these data sources is strongly influenced by effects on the early and moderate preterm population in which ACS constitute a powerful tool to prevent neonatal mortality, respiratory distress syndrome (RDS), intraventricular hemorrhage (IVH) and consecutive neurodevelopmental sequelae[9–11]. For this high risk population the overall protective effect from ACS on neurodevelopment is most probably achieved by the mitigation of immediate neonatal morbidity[11, 12]. However, given the low disease burden of late preterm infants (born at gestational age $34/0 - 36/6$) this evidence does not suffice for a treatment approach individualised by gestational age[13] and the majority of professional societies recommends against their use in this population[3–8]. Still late preterm infants suffer from increased neonatal morbidity [14–17] and impaired neurodevelopment, compared to their term born counterparts[18–21]. Similar to early preterm infants, impaired neurodevelopment seems to occur in the context of neonatal morbidity rather than gestational age alone[22]. Scince the ALPS trial was published in 2016[23] there is a growing body of evidence that respiratory morbidity can be mitigated by ACS administration after 34/0 weeks of gestation[24]. Still there are caveats about a general recommendation, as ACS on their own are supposed to have adverse effects on brain development through direct damage[25] as well as mediated by the increased risk for hypoglycemia[23, 24, 26–28]. Therefore the risk-benefit ratio of ACS strongly depends on the morbidity of the exposed population.

The ALPS trial excluded pregestational diabetes pregnancies, major fetal malformations, twin pregnancies and women who received any prior course of ACS, in order to prevent contamination of the control group[23]. It remains unclear weather or not recent ACS administrations in the late preterm period are superior to ACS administraions earlier in pregnancy. This question becomes important to clinical practioneers when caring for women with a relapsing risk for preterm birth after 34/0 weeks of gestation. The rate of ACS-exposed late preterms and term in-

fants constantly increased in the aftermath of the ALPS trial[29] despite ongoing concerns about neurologic longterm effects[30–35]. Therefore, the absence of external evidence about rescue-ACS administrations in the late preterm period might pose these infants at risk for repeated ACS-exposure in an uncontrolled environment with probably deleterious effects on the longterm neurologic outcome[31].

Data from deliveries before 34 weeks of gestation showed an optimal effect of ACS regarding the risk reduction of RDS when delivery occurs 2 to 7 days after ACS administration[36–42], but the risk reduction for longterm sequelae seems to extend beyond that period[37–41] − at least up to 10 days[43]. Thus the overall duration of protective ACS effects are difficult to determine. For ACS administration in the late preterm period there was no evidence for another increase of respiratory morbidity after 7 days[42]. Sudies on the effect of prior ACS administrations on the neonatal outcome in late preterm infants report conflicting results ranging from dramatic improvement to worsening of respiratory morbidity[44–47], but none of these studies reported the time interval from ACS administration to birth.

2 Goal

We conduct this pilot-study to evaluate the need for future trials on antenatal corticosteroid administration in pregnancies at relapsing risk for preterm birth in the late preterm period.

For this purpose we will compare the short term effects of antenatal betamethasone to the longterm effects of prior betamethasone administrations on the neonatal outcome in late preterm infants to identify relevant outcome parameters for further hypothesis-generation.

3 Question

Is there a difference in the neonatal outcome between late preterm infants (gestational age 34/0 − 36/6) after antenatal betamethasone exposure up to ten days prior birth and late preterm infants that received antenatal betamethasone more than 10 days prior birth with regard to death, cardiorespiratory complications, infection rates, short-term neurologic outcome, glucose metabolism, icterus, feeding, temperature regulation or hospital stay?

4 Design

4.1 Overview

We design this study as an observational cohort study. We will only use data already available from patient charts. We will not collect any additional data, nor will we extract personal data or make contact to former patients. We will abstain from any intervention that may influence the clinical decision making process on betamethasone administration or the neonatal outcome during the observed time period. With approval from the ethics-commitee of the Otto-von-Guericke University Magdeburg no additional patient consent other than given at hospital admission is needed. All women noted in the birth registry of the University Hospital for Gynecology, Obstetrics and Reproductive Medicine in Magdeburg, Germany from 01 January 2012 to 31 December 2018 will be screened for inclusion and exclusion criteria.

4.2 Inclusion Criteria

- Singleton Pregnancy
- Birth was given between 34/0 and 36/6 weeks of gestation
- At least one trial with 2 doses of intramuscular betmethasone 12 mg each was completed

4.3 Exclusion Criteria

- Insecure gestational age dating
- Unpredictable corticosteroid effects from...
  - ...incomplete betamethasone trial during pregnancy
  - ...dexamethasone administration during pregnancy
- Fetal malformation that may significantly alter the neonatal outcome
- Fetal inborn disease that may significantly alter the neonatal outcome

At our center antenatal betamethasone for fetal lung maturation is only administered up to 33/6 weeks of gestation by default, so we expect a large proportion of the cohort with recent

betamethasone administration to be of lower gestational age than the control group – probably not older than 35/2 weeks of gestation.

To make both cohorts comparable, the controls will be adjusted to make gestational age fit to the recent betamethasone cohort. At first this will be achieved by Restriction and, if necessary, by additional inverse probability weighting.

4.4 Cohort Assignment

We assing each mother–child diad to one of two cohorts. The Recent Betamethasone (RB) cohort contains all cases which completed the last betamethasone trial up to 10 days prior birth. The Past Betamethasone (PB) cohort contains all cases that completed the last betamethasone trial earlier than 10 days prior birth.

5 Item Selection

For all cases that fit the inclusion criteria in absence of any exclusion criteria the corresponding medical documentation are reviewed to extract the following items.

5.1 Maternal Data

Table 1: Maternal and Pregnancy Characteristics

**Maternal Age**	Difference between the infant's birth date and mother's birth date rounded down to full years
**Body Mass Index**	Maternal weight at pregnancy entry divided by the squared heigth in meters
**Gestational Age**	When the estimated birth date in the birth record matches the estimated birth date in the medical history, either derived from the first day of the last bleeding or from early ultrasound, compute Gestational Age (GA) from the estimated birth date.<br>In case of discordant information, seek confirmation for GA computed from either date in the doctor's letter and use this.<br>In case of discordant information from all three sources, check whether the estimated birth date from medical history fits the naegele rule (first day of last bleeding + 14 days, -3 months, + 1 year) or is derived from early ultrasound and use this. Only use the estimated birth date from early ultrasound if it deviates more than 14 days from the estimated birth date determined by history, when both are available.<br>In case none of the above applies, exclude the case for insecure gestational age dating.<br>In case information about the last bleeding or early ultrasound is missing or the gestational age dating is labled as insecure, exclude the case.
**Gravida**	Number of gestations as noted in the medical history
**Para**	Number of deliveries as noted in the medical history
**Miscarriages**	Number of miscarriages as noted in the medical history
**Abortions**	Number of abortions as noted in the medical history
**Stillbirths**	Number of stillbirths as noted in the medical history
**Caesarean Sections**	Number of prior caesarean sections as noted in the medical history
**Interval from last Bethamethasone**	Difference in days between the infant's birth date and the date of the second dose betamethasone during the last course.
**Gestational Age at Betamethasone**	Compute from the date of the second dose betamethasone during the last course and the estimated birth date as mentioned above.

Table 2: Pregnancy Risk Factors

**Gestational Age at Betamethasone**	Compute from the date of the second dose betamethasone during the last course and the estimated birth date as mentioned above.
**Caesarean Section**	Current pregnancy was terminated by caesarean section, documented in the mother's medical records.

**Assisted Reproduction**	Current pregnancy was induced by a medical intervention documented in the mother's medical history or the doctor's letter.
**Smoking in Pregnancy**	Smoking in pregnancy is noted in the medical history or the doctor's letter.
**Prior Pregnancy Complications**	Complications in any prior pregnancy are noted in the mother's medical history or the doctor's letter.
**Prior Birth Complications**	Birth complications in any prior pregnancy are noted in the mother's medical history or the doctor's letter.
**Pregnancy at Risk**	The examiner judges the current case to be at an increased risk for preterm delivery or increased neonatal morbidity.
**Medically Indicated Delivery**	Birth was indicated by the physicians team with regard to any medical condition in the mother and/or the fetus. This includes caesarean section as well as induced labour followed by vaginal delivery. In contrast premature rupture of membranes and premature labor in absence of any other medical condition are counted as Obstetrically Indicated Delivery.
**Gestational Diabetes**	Gestational diabetes or preexisting diabetes mellitus is noted in the mother's medical history or the doctor's letter.
**Insulin Therapy**	Insuline therapy for gestational diabetes or diabetes mellitus is noted in the mother's medical history or the doctor's letter.
**Makrosomia**	The neonate's actual birth weight exceeds the 90th percentile.
**Birth Arrest/ Mismatch**	Caesarean section was performed following a prolonged vaginal delivery attempt or medically indicated from malposition/malpresentation.
**Gestational Hypertonus**	Gestational Hypertonus is noted in the mother's medical history or the doctor's letter.
**Preeclampsia**	Preeclampsia is noted in the mother's medical history or the doctor's letter.
**HELLP-Syndrome**	HELLP-Syndrome is noted in the mother's medical history or the doctor's letter.
**Gestational Hepatosis**	Gestational hepatosis is noted in the mother's medical history or the doctor's letter.
**Intrauterine Growth Retardation**	Intrauterine growth retardation is noted in the mother's medical history AND the neonate's birth weight is below the 10th percentile.
**Prior Uterine Operation**	Any prior uterine operation, capable of leaving behind a transmural scar is noted in the mother's medical history or the doctor's letter.
**Uterine Malformation**	Any uterine malformation is noted in the mother's medical history or the doctor's letter.
**Chorionic Villous Sampling**	Any chorionic villous sampling was performed during the current pregnancy according to the mother's medical history or the doctor's letter.
**Bleeding in Pregnancy**	Bleeding has occured at any time during the current pregnancy and is noted in the mother's medical history or the doctor's letter.
**Strapping**	Strapping was performed in the current pregnancy and is noted in the mother's medical history or the doctor's letter.
**Anemia During Pregnancy**	At any time during the current pregnancy a hemoglobin level below 7.6 mmol/l was documented in the mother's laboratory documentation, or anemia is explicitly noted in the mother's medical history or the doctor's letter.
**Rhesus Conflict**	The mother's laboratory data is checked for rhesus status. In rhesus negative mothers the child's laboratory data is checked for rhesus status. If the child is rhesus positive the case is counted as conflicting rhesus status.
**Coombs Test Positive**	In cases counted as conflicting rhesus status the child's laboratory data is checked for the coombs test result. Cases with positive coombs test results are counted.

**Suspected Triple I**	Counted if birth records contain body temperature measurements >39°C or repeated measurements >38°C PLUS at least one of the following: • the cardiotocogram shows fetal tachykardia >160 bpm for >10 minutes • the maternal leukocyte count is >15 Gpt/l prior birth • purulent cervical discharge is noted by the staff
**Confirmed Triple I**	Counted if the criteria for Suspected Triple I are met (see above) AND placental histology shows signs of placental or chorioamniotic inflammation. Amniotic fluid samples are not usually conducted at our site, but positive gram stain, positive amniotic fluid culture, leucocyte count >30/mm^3 or Glucose <0,78 mmol/l in the amniotic fluid may be taken as substitutes, if available.
**STORCHL-Infection**	Counted if the laboratory data and/or history document any vertically transmissible infection during the current pregnancy.
**Streptococcus B Colonization**	Counted if laboratory data confirms vaginal colonisation with Streptococcus B species.
**Bleeding Immediatly Prior to Birth**	Counted if bleeding occured immediately prior to birth or partial/total premature placental abruption occured.
**Patial Premature Placental Abruption**	Counted if partial premature placental abruption is documented.
**Premature Placental Abruption**	Counted if total premature placental abruption is documented.

5.2 Infant Data

Table 3: Neonatal characteristics

**Birthweight**	Read the neonate's birthweight in [g] from the birth record. If not available there, read the birthweight from the neonatology admission papers or use the first weight documented in the patient's medical chart. Don't use data that was measured after the 5th day of life.
**Birthweight Percentile**	Calculate the birthweight percentile and z-score from Birthweight and gestational age at the time of measurement using the data from Voigt 2006[48]
**Length at Birth**	Read the neonate's length in [cm] from the birth record. If not available there, read the length from the neonatology admission papers or use the first length documented in the patient's medical chart. Don't use data that was measured after the 5th day of life.
**Length at Birth - percentile**	Calculate the length percentile and z-score from Length at Birth and gestational age at the time of measurement using the data from Voigt 2006[48]
**Head Circumference at Birth**	Read the neonate's head circumference in [cm] from the birth record. If not available there, read the head circumference from the neonatology admission papers or use the first head circumference documented in the patient's medical chart. Don't use data that was measured after the 5th day of life.
**Head Circumference at Birth - percentile**	Calculate the head circumference percentile and z-score from Head Circumference at Birth and gestational age at the time of measurement using the data from Voigt 2006[48]
**Gender**	Read the neonate's gender phenotype from the birth record. If not available, use the first documented gender phenotype from clinical examinations.

**Small For Gestational Age**	Count if Birthweight Percentile is <10

Table 4: Neonatal Outcome - Death

**Stillbirth**	Count if the current pregnancy ended in a stillbirth.
**Death During First 72h of Life**	Count if the neonate died within 72 hours of life.
**Death During Neonatal Period**	Count if the neonate died within 28 days of life.

Table 5: Neonatal Outcome - Cardiorespiratory

**Supported Transition**	Count if the neonate needed respiratory support during the first 30 minutes of life.
**Respirtory Transition Disorder**	Count if the neonate showed signs of respiratory distress during the first 2h of life.
**Intubation**	Count if the neonate needed endotracheal ventilation anytime during the hospital stay.
**High Flow Nasal Cannula**	Count if the neonate received Highflow Nasal Cannula ventilation anytime during the hospital stay.
**Continuous Positive Airway Pressure**	Count if the neonate received Continuous Positive Airway Pressure ventilation anytime during the hospital stay.
**Inspired Oxygen Fraction >0.3**	Count if the neonate received ventilation with more than 0.3 inspired oxygen fraction anytime during the hospital stay.
**Any respiratory support**	Count if the neonate received any respiratory support during the hospital stay.
**CPAP/HFNC >2h**	Count if the neonate received more than 2 hours of Continuous Positive Airway Pressure ventilation in total during the hospital stay.
**CPAP/HFNC >12h**	Count if the neonate received more than 12 hours of Continuous Positive Airway Pressure ventilation in total during the hospital stay.
**FiO2 >0.3 longer than 4h**	Count if the neonate received ventilation with an inspired oxygen fraction >0.3 for more than 4 hours of in total during the hospital stay.
**FiO2 >0.3 longer than 24h**	Count if the neonate received ventilation with an inspired oxygen fraction >0.3 for more than 24 hours of in total during the hospital stay.
**Extracorporal Membrane Oxygenation**	Count if the neonate received extracorporal membrane oxygenation anytime during the hospital stay.
**Surfactant**	Count if the neonate received endotracheal surfactant application anytime during the hospital stay.
**Apnoe**	Count if the neonate was treated with caffeine citrate for relevant apnoea episodes.
**Pneumothorax**	Count if the neonate was recognised to have pneumothorax with or without drainage.
**Mekonium Aspiration Syndrome**	Count if the neonate was labled with mekonium aspiration syndrome in the doctor's letter.

**Bronchopulmonary Dysplasia**	Count if the neonate received any form of respiratory support or supplemental oxygen for more than 672 hours (28 days).
**PPHN**	Count if the neonate was labled with persistent pulmonary hypertension in echocardiography findings.
**Persistent Fetal Circulation**	Count if the neonate was labled with persistent pulmonary hypertension in echocardiography findings and had a right to left shunt over the ductus arteriosus at the same time.
**Arterial Hypotension**	Count if the neonate was treated with additional volume and/or cathecholamines for arterial hypotension.
**Hemodynamically Relevant PDA**	Count if the neonate was recognised to have a hemodynamically relevant persistent ductus arteriosus in echocardiography.
**PDA: Ibuprofene Therapy**	Count if the neonate was recognised to have a hemodynamically relevant persistent ductus arteriosus in echocardiography and was treated with Ibuprofene.
**PDA: Ibuprofene Therapy**	Count if the neonate was recognised to have a hemodynamically relevant persistent ductus arteriosus in echocardiography and was treated with Ibuprofene.
**Umbilical Artery pH**	Read the umbilical artery pH from the laboratory data.
**Umbilical Vein pH**	Read the umbilical artery pH from the laboratory data.
**APGAR 1'**	Read the one-minute APGAR-Score from the birth record. If not available there, read it from the resuscitation protocol from the neonatology department.
**APGAR 5'**	Read the five-minute APGAR-Score from the birth record. If not available there, read it from the resuscitation protocol from the neonatology department.
**APGAR 10'**	Read the ten-minute APGAR-Score from the birth record. If not available there, read it from the resuscitation protocol from the neonatology department.
**Duration of Mechanical Ventilation**	Read the time in minutes for mechanical ventilation from the resuscitation protocol (1-minute intervals) and the neonate's medical record (5-minute intervals) from intubation to extubation and calculate it into decimal hours.
**CPAP Duration**	Read the time in minutes for continuous positive airway pressure (CPAP) support from the resuscitation protocol (1-minute intervals) and the neonate's medical record (5-minute intervals) and calculate it into decimal hours.
**HFNC Duration**	Read the time in minutes for high-flow nasal cannula (HFNC) support from the neonate's medical record (5-minute intervals) and calculate it into decimal hours.
**Overall Need for Respiratory Support**	Sum up the durations of mechanical ventilation, CPAP-support and HFNC-support
**Duration of FiO_2 >0.3**	Read the time in minutes for any respiratory support or low-flow oxygen supplementation with an inspiratory oxygen fraction of 0.3 or more from the resuscitation protocol (1-minute intervals) and the neonate's medical record (5-minute intervals) and calculate it into decimal hours.
**Treatment with Caffeine in Days**	Count the days with caffeine treatment as noted in the neonate's medical record.
**Need for Volume Administration**	Count the numbers of additional volume administration (20ml/kg) rounded up to full integers, as noted in the neonate's medical record.
**Need for Catecholamine Treatment**	Count the days with catecholamine administration noted in the neonate's medical record.

Table 6: Neonatal Outcome - Infection

**Antibiotic Therapy**	Count if the neonate was treated with anibiotics according to the medical record.
**Neonatal Infection/Sepsis**	Count the neonate was diagnosed with bacterial infection and/or sepsis according to the doctor's letter.
**Pneumonia**	Count the neonate was diagnosed with Pneumonia according to the doctor's letter.
**Necrotizing Enterocolitis**	Count the neonate was diagnosed with necrotizing enterocolitis (NEC) according to the doctor's letter.

Table 7: Neonatal Outcome - Neurologic

**Asphyxia**	Count if the umbilical artery pH was <7.00 or umbilical artery base excess was ≤ 12 mmol/l or 5'APGAR was ≤ 5. Cases with stillbirth are excluded from this category.
**Therapeutic Hypothermia**	Count if the neonate was treated with therapeutic hypothermia following asphyxia.
**Neurologic Sequelae**	Count if the neonate was diagnosed with any condition of persistent neurologic damage (i.E. periventricular leukomalacia, intracerebral bleeding, cerebral infarction, hypoxic ischemic encephalopathy, cerebral palsy and others).
**Subependymal Hemorrhage**	Count if the neonate was diagnosed with subependymal hemorrhage according to cerebral ultrasound findings (IVH Grade I).
**Intraventtricular Hemorrhage Grade II**	Count if the neonate was diagnosed with intraventricular hemorrhage grade II according to cerebral ultrasound findings (IVH Grade II).
**Intraventtricular Hemorrhage Grade III**	Count if the neonate was diagnosed with intraventricular hemorrhage grade III according to cerebral ultrasound findings (IVH Grade III).

Table 8: Neonatal Outcome - Metabolic

**Early Feeding**	Count if the neonate received the first feeding up to 30 minutes after birth.
**Hypoglycemia**	Count if any blood glucose level <2.5 mmol/l was detected in the neonate according to the laboratory data.
**Severe Hypoglycemia**	Count if any blood glucose level <1.8 mmol/l was detected in the neonate according to the laboratory data or any blood glucose level <2.5 mmol/l was temporally associated with neurologic and/or vegetative symptoms according to the medical record.
**Carbohydrate Substitution**	Count if the neonate received any oral or intravenous carbohydrate substitution.
**Intravenous Glucose Substitution**	Count if the neonate received any gucose containing i.v. solution.
**Umbilical Vessel Glucose**	Read the umbilical artery glucose level in mmol/l from the laboratory data. If umbilical artery data is not available use the umbilical vein glucose level.
**Lowest Blood Glucose**	Identify the lowest blood glucose level in mmol/l from the neonate's laboratory data, excluding the umbilical vessel glucose level.
**Glucose i.v. - Parenteral Feeding**	Count the days of parenteral feeding with glucose containing i.v. solutions in the neonate's medical record. Do not count these days in another Glucose i.v. category other than "- Overall".

**Glucose i.v. - Hypoglycemia Therapy**	Count the days with glucose containing i.v. solutions following an event of hypoglycemia in the neonate's medical record. Do not count these days in another Glucose i.v. category other than "- Overall".
**Glucose i.v. - Other Cause**	Count the days with glucose containing i.v. solutions in the neonate's medical record that was indicated independent from feeding or hypoglycemia (i.E. intravenous access protection for ongoing i.v. therapy). Do not count these days in another Glucose i.v. category other than "- Overall".
**Glucose i.v. - Overall**	Sum up the days with any glucose containing i.v. solution in the neonate's medical record.

Table 9: Neonatal Outcome - Icterus

**Polyglobulia**	Count if the neonate's hematocrite exceeded 0.65 in the laboratory data at any point in time.
**Hyperbilirubinemia**	Count if the neonate received any therapy for hyperbilirubinemia.
**Highest Bilirubin**	Identify the highest bilirubin level in µmol/l from the neonate's medical record (tc-bilirubin) and the neonate's laboratory data (total-bilirubin from capillary or venous blood samples). Do not include tc-bilirubin data from days where total-bilirubin data is available.
**Highest Bilirubin**	Identify the highest bilirubin level in µmol/l from the neonate's medical record (tc-bilirubin) and the neonate's laboratory data (total-bilirubin from capillary or venous blood samples). Do not include tc-bilirubin data from days where total-bilirubin data is available.
**Phototherapy**	Count the phototherapy cycles (24h intervals) the neonate received according to the neonate's medical record.
**Blood Exchange**	Count the blood exchange cycles the neonate received according to the neonate's medical record.

Table 10: Neonatal Outcome - Feeding

**Latency to First Feeding**	Calculate the time in minutes between birth according to the birth record and the first feeding according to the neonate's medical record.
**Latency to First Own Mother's Milk**	Count the days without own mother's milk (OMM) feeding beginning at the day of birth until the first OMM feeding occured. Exclude cases with ablactation.
**Additional Formula Feds**	Count the days with formula feds from the day of first OMM-feeding to discharge. Exclude cases with ablactation.
**Days Until Enteral Feeding Only**	Count the days from birth to the last day of parenteral feeding as noted in the neonate's medical record.
**Gastric tube feeding**	Count the days from birth to the last day of gastric tube feeding as noted in the neonate's medical record.
**Gastroesophageal Reflux**	Count the days with overt gastroesophageal reflux noted in the neonate's medical record.
**Ablactation**	Count if ablactation was noted in the mother's medical record.
**Additional Formula Feeding at Discharge**	Count if the neonate received additional formula feds up to 24h before discharge.

Table 11: Neonatal Outcome - Temperature Regulation

**Temperature Regulation Disorder**	Count if the neonate received any form of external heat.
**Incubator**	Count the days the neonate spent in an incubator with capabilities of external heat and humidity regulation according to the neonate's medical record. Do not count days that are already counted as "Warm Bed/Heat Lamp" days.
**Warm Bed/Heat Lamp**	Count the days the neonate received external heat outside an incubator as defined above, either from a warm bed as used at the neonatology ward or under a heat lamp as used at the maternity ward. Do not count days that are already counted as "Incubator" days.
**Overall Need for External Heat**	Sum up the days the neonate received external heat from any source.

Table 12: Neonatal Outcome - Hospital Stay

**Rooming In on the 1st Day of Life**	Count if the neonate was admitted to the maternity ward immediately after birth.
**Neonatal Intensive Care Unit Stay**	Count if the neonate was admitted to the neonatal intensive care unit (NICU) anytime during the hospital stay.
**Lenght of Hospital Stay >21 Days**	Count if the neonate stayed in hospital for more than 21 days.
**Discharge at Gestational Age >40/0**	Count if the neonate was discharged from hospital after the estimated birth date.
**NICU Stay**	Count the days the neonate spent on the NICU according to the neonate's medical record.
**Hospital Stay**	Calculate the days the neonate spent in hospital from birth date and date of dismission according to the doctor's letter. In case of transfer to another hospital cap this number at the date of transfer and note the risk for bias (see below).
**Gestational Age at Discharge**	Calculate gestational age at discharge from the estimated birth date and the date of discharge according to the doctor's letter.
**Weight at Discharge**	Read the weight at the day of discharge from the neonate's medical record.

5.3 Risk for BIAS

Table 13: Risk for BIAS

**Transfer to Hospital Close to Home Prior Discharge**	Count as a risk for bias if the neonate was transferred to a hospital close to home due to advanced maturity.
**Discharge Against Physician's Advice**	Count as a risk for bias if the neonate was dismissed prematurely against the physician's advice.
**Incomplete Documentation**	Count as a risk for bias if relevant medical documents or parts of them are missing.

5.4 Composite Outcomes

To better display the occurence of relevant events we generate composite outcome parameters out of the primary items above.

Table 14: Composite Outcome Parameters

**Composite Outcome - Death**	Defined as stillbirth or neonatal death up to 28 days of life.
**Composite Outcome - Cardiorespiratory**	Defined as any of the following:<br>• CPAP or HFNC longer than 2 hours in sum<br>• Inspired oxygen fraction (FiO2) >0.3 longer than 4 hours<br>• surfactant application<br>• need for mechanical ventilation<br>• external membrane oxygenation (ECMO)<br>• pneumothorax<br>• bonchopulmonal dysplasia (BPD)<br>• arterial hypotension
**Composite Outcome - Neurologic**	Defined as the incidence of any intracranial bleeding, encephalopathy or neonatal seizures.
**Composite Outcome - Metabolic**	Defined as either severe hypoglycemia or the need for intravenous glucose infusion.
**Composite Outcome - Feeding**	Defined as either ablactation or need for additional formula feds at dismission/transfer to another hospital.
**Composite Outcome - Temperature Reguation**	Defined as either need for external heat or the lack of rooming in on the first day of life.
**Composite Outcome - Hospital Stay**	Defined as Neonatal Intensive Care Unit (NICU) stay of any duration, hospital stay >21 days or transfer to another hospital close to home (lost to followup).
**Composite Outcome - Combined**	Defined as the occurence of any Composite Outcome as mentioned above.
**Composite Outcome - BIAS**	Defined as any of the following:<br>• Transfer to Hospital Close to Home Prior Discharge (lost to followup)<br>• Discharge Against Physician's Advice<br>• Incomplete Documentation

6 Analysis

- Data will be grouped into dicotomous and continuous data.
- Dicotomous variables will be compared between both cohorts using fisher's test.
- Dicotomous variables will be reported as whole numbers, percent of total in brackets, odd's ratio and p-value.
- Continuous data will be compared between both cohorts using the brunner-munzel test.
- Continuous data will be reported as median, range in brackets, Δmean and p-value.
- Percentiles will be converted into z-scores before analysis.
- Pregnancy risk profiles will be compared first to ensure comparability of both cohorts, if risk profiles diverge appropriate adjustments will be conducted to ensure comparability between both cohorts and to reduce selection bias.
- All items and composite outcome parameters will be compared between the final cohorts
- We will model a high-risk subgroup by selection of those patients that needed any form of respiratory support and compare the cardiorespiratory items between the subgroup-cohorts
- Due to the retrospective design we expect a low rate of cases to be lost to followup. Data is included as available and the risk of bias from cases lost to followup will be reported. Exclusion will occur only if the bias is judged to be relevant.
- To suit the purpose of a pilot study we aim to characterise our cohorts as good as possible. Therefore we analyse multiple items per category that may have an influence on the neonatal outcome. We are aware of the risk for a cumulating $\alpha-$error and possible false-positive findings. In part this risk will be mitigated when data interpretation focuses only on the composite outcome parameters as indicators for relevant adverse outcomes in each category. We accept the risk of $\alpha-$error accumulation, because our results are only meant to serve for further hypothesis generation.

7 Funding and Conflicting Interests

We do not receive any external funding for this study. We declare to have no conflicting interest.

References

1. Liggins GC and Howie RN. A Controlled Trial of Antepartum Glucocorticoid Treatment for Prevention of the Respiratory Distress Syndrome in Premature Infants. Pediatrics 1972;50:515–25.

2. Obstetricians {and} Gynecologists AAC of. ACOG Committee Opinion No. 713: Antenatal Corticosteroid Therapy for Fetal Maturation. Obstet Gynecol 2017;130:e102–e9.

3. Liggins Institute The University of Auckland ANZ. Antenatal corticosteroids given to women prior to birth to improve fetal, infant, child and adult health: Clinical Practice Guidelines. Clinical Practice Guideline. Liggins Institute, The University of Auckland, Auckland. New Zealand.: Antenatal Corticosteroid Clinical Practice Guidelines Panel., 2015.

4. Skoll A, Boutin A, Bujold E, et al. SOGC Clinical Practice Guideline No. 364-Antenatal Corticosteroid Therapy for Improving Neonatal Outcomes. Journal of obstetrics and gynaecology Canada: JOGC = Journal d'obstetrique et gynecologie du Canada: JOGC 2018;40:1219–39.

5. AWMF. Prevention and therapy of preterm labour. Guideline of the DGGG, OEGGG and SGGG (S2k-Level, AWMF Registry No.015/025, February 2019). Registry No.015/025. DGGG, OEGGG and SGGG, 2019:228.

6. Organization WH. WHO recommendations on antenatal corticosteroids for improving preterm birth outcomes. WHO Guidelines Approved by the Guidelines Review Committee. Geneva: World Health Organization, 2022. URL: http://www.ncbi.nlm.nih.gov/books/NBK585366/ (visited on 03/06/2023).

7. Health {and} Care Excellence NNI for. Preterm labour and birth. NICE Guideline [NG25]. Publisher: NICE. NICE. National Institute for Health and Care Excellence, 2022. URL: https://www.nice.org.uk/guidance/ng25/chapter/Recommendations#mode-of-birth (visited on 03/06/2023).

8. Stock S, Thomson A, Papworth S, and the Royal College of Obstetricians and Gynaecologists. Antenatal corticosteroids to reduce neonatal morbidity and mortality: Green-top

Guideline No. 74. BJOG: An International Journal of Obstetrics & Gynaecology 2022;129. _eprint: https://onlinelibrary.wiley.com/doi/pdf/10.1111/1471-0528.17027:e35–e60.

9. Roberts D and Dalziel SR. Antenatal corticosteroids for accelerating fetal lung maturation for women at risk of preterm birth. Cochrane Database of Systematic Reviews 2006. In collab. with The Cochrane Collaboration:CD004454.pub2.

10. Roberts D, Brown J, Medley N, and Dalziel SR. Antenatal corticosteroids for accelerating fetal lung maturation for women at risk of preterm birth. The Cochrane Database of Systematic Reviews 2017;3:CD004454.

11. McGoldrick E, Stewart F, Parker R, and Dalziel SR. Antenatal corticosteroids for accelerating fetal lung maturation for women at risk of preterm birth. Cochrane Database of Systematic Reviews 2020. Publisher: John Wiley & Sons, Ltd.

12. Sotiriadis A, Tsiami A, Papatheodorou S, Baschat AA, Sarafidis K, and Makrydimas G. Neurodevelopmental Outcome After a Single Course of Antenatal Steroids in Children Born Preterm: A Systematic Review and Meta-analysis. Obstetrics and Gynecology 2015;125:1385–96.

13. Groom KM. Antenatal corticosteroids after 34 weeks' gestation: Do we have the evidence? Seminars in Fetal and Neonatal Medicine 2019;24. Publisher: Elsevier:189–96.

14. McIntire DD and Leveno KJ. Neonatal Mortality and Morbidity Rates in Late Preterm Births Compared With Births at Term. Obstetrics & Gynecology 2008;111.

15. Yoder BA, Gordon MC, and Jr WHB. Does the Changing Obstetric Paradigm Alter the Epidemiology of Respiratory Complications? Obstetrics & Gynecology 2008;111.

16. Hibbard JU, Wilkins I, Sun L, et al. Respiratory morbidity in late preterm births. JAMA 2010;304:419–25.

17. Boyle EM, Johnson S, Manktelow B, et al. Neonatal outcomes and delivery of care for infants born late preterm or moderately preterm: a prospective population-based study. Archives of Disease in Childhood. Fetal and Neonatal Edition 2015;100:F479–485.

18. Vohr B. Long-term outcomes of moderately preterm, late preterm, and early term infants. Clinics in Perinatology 2013;40:739–51.

19. Walsh JM, Doyle LW, Anderson PJ, Lee KJ, and Cheong JLY. Moderate and late preterm birth: effect on brain size and maturation at term-equivalent age. Radiology 2014;273:232–40.

20. Chan E and Quigley MA. School performance at age 7 years in late preterm and early term birth: a cohort study. Archives of Disease in Childhood. Fetal and Neonatal Edition 2014;99:F451–457.

21. Mirzakhani H, Kelly RS, Yadama AP, et al. Stability of developmental status and risk of impairment at 24 and 36 months in late preterm infants. Infant Behavior & Development 2020;60:101462.

22. Baron IS, Erickson K, Ahronovich MD, Litman FR, and Brandt J. Spatial location memory discriminates children born at extremely low birth weight and late-preterm at age three. Neuropsychology 2010;24:787–94.

23. Gyamfi-Bannerman C, Thom EA, Blackwell SC, et al. Antenatal Betamethasone for Women at Risk for Late Preterm Delivery. New England Journal of Medicine 2016;374:1311–20.

24. Saccone G and Berghella V. Antenatal corticosteroids for maturity of term or near term fetuses: systematic review and meta-analysis of randomized controlled trials. BMJ (Clinical research ed.) 2016;355:i5044.

25. Merwe JL van der, Sacco A, Toelen J, and Deprest J. Long-term neuropathological and/or neurobehavioral effects of antenatal corticosteroid therapy in animal models: a systematic review. Pediatric Research 2020;87:1157–70.

26. Kerstjens JM, Bocca-Tjeertes IF, Winter AF de, Reijneveld SA, and Bos AF. Neonatal morbidities and developmental delay in moderately preterm-born children. Pediatrics 2012;130:e265–272.

27. McKinlay CJD, Alsweiler JM, Anstice NS, et al. Association of Neonatal Glycemia With Neurodevelopmental Outcomes at 4.5 Years. JAMA pediatrics 2017;171:972–83.

28. Shah R, Harding J, Brown J, and McKinlay C. Neonatal Glycaemia and Neurodevelopmental Outcomes: A Systematic Review and Meta-Analysis. Neonatology 2019;115:116–26.

29. Kearsey EOR, Been JV, Souter VL, and Stock SJ. The impact of the Antenatal Late Preterm Steroids trial on the administration of antenatal corticosteroids. American Journal of Obstetrics and Gynecology 2022;227:280.e1–280.e15.

30. Stutchfield PR, Whitaker R, Gliddon AE, Hobson L, Kotecha S, and Doull IJM. Behavioural, educational and respiratory outcomes of antenatal betamethasone for term caesarean section (ASTECS trial). Archives of Disease in Childhood. Fetal and Neonatal Edition 2013;98:F195–200.

31. Asztalos E, Willan A, Murphy K, et al. Association between gestational age at birth, antenatal corticosteroids, and outcomes at 5 years: multiple courses of antenatal corticosteroids for preterm birth study at 5 years of age (MACS-5). BMC pregnancy and childbirth 2014;14:272.

32. Melamed N, Asztalos E, Murphy K, et al. Neurodevelopmental disorders among term infants exposed to antenatal corticosteroids during pregnancy: a population-based study. BMJ open 2019;9:e031197.

33. Räikkönen K, Gissler M, and Kajantie E. Associations Between Maternal Antenatal Corticosteroid Treatment and Mental and Behavioral Disorders in Children. JAMA 2020;323:1924–33.

34. Lin YH, Lin CH, Lin MC, Hsu YC, and Hsu CT. Antenatal corticosteroid exposure is associated with childhood mental disorders in late preterm and term infants. The Journal of Pediatrics 2022:S0022–3476(22)00872–1.

35. Sarid EB, Stoopler ML, Morency AM, and Garfinkle J. Neurological implications of antenatal corticosteroids on late preterm and term infants: a scoping review. Pediatric Research 2022;92:1225–39.

36. Sekhavat L, Firouzabadi RD, and Karbasi SA. Comparison of interval duration between single course antenatal corticosteroid administration and delivery on neonatal outcomes. Journal of the Turkish German Gynecological Association 2011;12:86–9.

37. Peaceman AM, Bajaj K, Kumar P, and Grobman WA. The interval between a single course of antenatal steroids and delivery and its association with neonatal outcomes. American Journal of Obstetrics and Gynecology 2005;193:1165–9.

38. Battarbee AN, Ros ST, Esplin MS, et al. Optimal timing of antenatal corticosteroid administration and preterm neonatal and early childhood outcomes. American journal of obstetrics & gynecology MFM 2020;2:100077.

39. Biedermann R, Schleussner E, Lauten A, et al. Inadequate Timing Limits the Benefit of Antenatal Corticosteroids on Neonatal Outcome: Retrospective Analysis of a High-Risk Cohort of Preterm Infants in a Tertiary Center in Germany. Geburtshilfe Und Frauenheilkunde 2022;82:317–25.

40. Fortmann I, Mertens L, Boeckel H, et al. A Timely Administration of Antenatal Steroids Is Highly Protective Against Intraventricular Hemorrhage: An Observational Multicenter Cohort Study of Very Low Birth Weight Infants. Frontiers in Pediatrics 2022;10:721355.

41. Wang D, Ming L, and Zhu Y. Antenatal corticosteroid administration-to-birth interval and neonatal outcomes in very preterm infants: A secondary analysis based on a prospective cohort study. PloS One 2023;18:e0281509.

42. Gulersen M, Gyamfi-Bannerman C, Greenman M, Lenchner E, Rochelson B, and Bornstein E. Time interval from late preterm antenatal corticosteroid administration to delivery and the impact on neonatal outcomes. American journal of obstetrics & gynecology MFM 2021;3:100426.

43. Liebowitz M and Clyman RI. Antenatal Betamethasone: A Prolonged Time Interval from Administration to Delivery Is Associated with an Increased Incidence of Severe Intraventricular Hemorrhage in Infants Born before 28 Weeks Gestation. The Journal of Pediatrics 2016;177:114–120.e1.

44. Gyamfi-Bannerman C, Gilbert S, Landon MB, et al. Effect of antenatal corticosteroids on respiratory morbidity in singletons after late-preterm birth. Obstetrics and Gynecology 2012;119:555–9.

45. Eriksson L, Haglund B, Ewald U, Odlind V, and Kieler H. Health consequences of prophylactic exposure to antenatal corticosteroids among children born late preterm or term. Acta Obstetricia et Gynecologica Scandinavica 2012;91:1415–21.

46. Ventolini G, Neiger R, Mathews L, Adragna N, and Belcastro M. Incidence of respiratory disorders in neonates born between 34 and 36 weeks of gestation following exposure to antenatal corticosteroids between 24 and 34 weeks of gestation. American Journal of Perinatology 2008;25:79–83.

47. Arimi Y, Zamani N, Shariat M, and Dalili H. The effects of betamethasone on clinical outcome of the late preterm neonates born between 34 and 36 weeks of gestation. BMC pregnancy and childbirth 2021;21:774.

48. Voigt M, Fusch C, Olbertz D, et al. Analyse des Neugeborenenkollektivs der Bundesrepublik Deutschland. Geburtshilfe und Frauenheilkunde 2006;66:956–70.
