# Supplementary material for: The influence of antenatal betamethasone timing on neonatal outcome in late preterm infants: a single-center cohort study
Source: Arch Gynecol Obstet. 2024 Sep 9;311(4):1017–27. doi: 10.1007/s00404-024-07714-9 (PMC11985642; doi:10.1007/s00404-024-07714-9)
Supplement: Supplementary file 2 — (pdf 0 KB) [file 404_2024_7714_MOESM2_ESM.pdf]

Archives of Gynecology and Obstetrics: The Influence of Antenatal Betamethasone Timing on Neonatal Outcome in Late Preterm Infants Supplemental Information: Tables

Thomas Brückner[1*] and Anke Redlich[2]

[1*]Paediatrics, Medical Faculty, Otto-von-Guericke University, Leipziger Str. 44, Magdeburg, 39120, Sachsen-Anhalt, Germany.
[2]University Hospital for Obstetrics and Gynecology, Medical Faculty, Otto-von-Guericke University, Gerhart-Hauptmann Straße 35, Magdeburg, 39108, Sachsen-Anhalt, Germany.

*Corresponding author(s). E-mail(s): thomas.brueckner@charite.de;
Contributing authors: anke.redlich@med.ovgu.de;

*Present Address:
Charité - Universitätsmedizin Berlin, SPZ-Neuropädiatrie, Augustenburger Platz 1, Campus: Ostring 1, 13353 Berlin, Germany.

List of Tables

1 Original Maternal and Pregnancy Characteristics 3
2 Corrected Maternal and Pregnancy Characteristics 4
3 Pregnancy Risk Profiles 5
4 Neonatal Characteristics 6
5 Neonatal Outcome - Death 7
6 Neonatal Outcome - Cardiorespiratory 8
7 Subgroup Analysis - Neonates With Adverse Respiratory Outcome . . 9
8 Neonatal Outcome - Infection 10
9 Neonatal Outcome - Neurologic 11
10 Neonatal Outcome - Metabolic 12
11 Neonatal Outcome - Icterus 13
12 Neonatal Outcome - Feeding 14
13 Neonatal Outcome - Temperature Regulation 15
14 Neonatal Outcome - Hospital Stay 16
15 Neonatal Outcome - Composite Outcomes 17
16 Sources of BIAS 18

Table 1: Original Maternal and Pregnancy Characteristics

	**Recent Betamethasone** median	(range) [n=8]	**Past Betamethasone** median	(range) [n=126]	Δmean	p-value
Maternal Age [a]	28.5	(22 - 36)	30	(15 - 41)	-1.00	0.518
Body Mass Index [kg/m^2]	25.7	(15.1 - 29.4)	23.75	(16.7 - 47.8)	-0.96	0.899
Gestational Age [d]	240.5	(239 - 252)	248.5	(238 - 258)	-5.15	0.017
Gravida [n]	2	(1 - 4)	2	(1 - 7)	-0.04	0.776
Para [n]	1	(1 - 3)	1	(1 - 5)	-0.13	0.606
Miscarriages [n]	1	(0 - 1)	0	(0 - 3)	0.30	0.090
Abortions [n]	0	(0 - 0)	0	(0 - 3)	-0.19	<0.001
Stillbirths [n]	0	(0 - 0)	0	(0 - 1)	-0.02	0.083
Cesarean Sections [n]	1	(0 - 3)	1	(0 - 4)	0.12	0.650
Latency to Bethamethasone [d]	7.5	(0 - 10)	40	(11 - 98)	-37.90	<0.001
GA at Betamethasone [d]	235.5	(230 - 252)	208.5	(160 - 236)	32.46	<0.001

Table 2: Corrected Maternal and Pregnancy Characteristics

	**Recent Betamethasone**		**Past Betamethasone**			
	median	(range) [n=8]	median	(range) [n=89]	Δmean	p-value
Maternal Age [a]	28.5	(22 - 36)	30	(17 - 41)	-0.93	0.558
Body Mass Index [kg/m²]	25.7	(15.1 - 29.4)	23.1	(17.9 - 42.3)	-0.86	0.798
Gestational Age [d]	240.5	(239 - 252)	244	(238 - 252)	-1.98	0.303
Gravida [n]	2	(1 - 4)	2	(1 - 7)	0.00	0.652
Para [n]	1	(1 - 3)	1	(1 - 5)	-0.17	0.550
Miscarriages [n]	1	(0 - 1)	0	(0 - 2)	0.37	0.077
Abortions [n]	0	(0 - 0)	0	(0 - 3)	-0.17	<0.001
Stillbirths [n]	0	(0 - 0)	0	(0 - 1)	-0.01	0.320
Cesarean Sections [n]	1	(0 - 3)	1	(0 - 4)	0.12	0.675
Latency to Bethamethasone [d]	7.5	(0 - 10)	34	(11 - 80)	-33.73	<0.001
GA at Betamethasone [d]	235.5	(230 - 252)	209	(170 - 236)	31.39	<0.001

Table 3: Pregnancy Risk Profiles

	Recent Betamethasone		Past Betamethasone			
	no.	(%) [n=8]	no.	(%) [n=89]	odds ratio	p-value
Cesarian Section	7	(87.5)	56	(62.9)	4.08	0.254
Assisted Reproduction	0	(0.0)	7	(7.9)	0.00	1.000
Smoking in Pregnancy	2	(25.0)	19	(21.3)	1.23	1.000
Prior Pregnancy Complications	1	(12.5)	26	(29.2)	0.35	0.437
Prior Birth Complications	1	(12.5)	22	(24.7)	0.44	0.676
Pregnancy at Risk	8	(100.0)	86	(96.6)	Inf	1.000
Medically Indicated Delivery	7	(87.5)	43	(48.3)	7.36	0.060
Gestational Diabetes	0	(0.0)	15	(16.9)	0.00	0.351
Insulin Therapy	0	(0.0)	7	(7.9)	0.00	1.000
Makrosomia	0	(0.0)	1	(1.1)	0.00	1.000
Birth Arrest/Mismatch	0	(0.0)	10	(11.2)	0.00	1.000
Gestational Hypertonus	1	(12.5)	6	(6.7)	1.96	0.464
Preeclampsia	0	(0.0)	7	(7.9)	0.00	1.000
HELLP-Syndrome	1	(12.5)	4	(4.5)	2.98	0.356
Gestational Hepatosis	0	(0.0)	0	(0.0)	0.00	1.000
Intrauterine Growth Retardation	3	(37.5)	18	(20.2)	2.34	0.365
Prior Uterine Operation	1	(12.5)	26	(29.2)	0.35	0.437
Uterine Malformation	0	(0.0)	5	(5.6)	0.00	1.000
Chorionic Villus Sampling	0	(0.0)	1	(1.1)	0.00	1.000
Bleeding in Pregnancy	0	(0.0)	25	(28.1)	0.00	0.108
Strapping	0	(0.0)	3	(3.4)	0.00	1.000
Anemia During Pregnancy	5	(62.5)	50	(56.2)	1.30	1.000
Rhesus Incompatibility	1	(12.5)	6	(6.7)	1.96	0.464
Coombs Test Positive	1	(33.3)	1	(12.5)	3.06	0.491
Amiotic Infection Syndrome	0	(0.0)	1	(1.1)	0.00	1.000
Suspectet Triple I	0	(0.0)	0	(0.0)	0.00	1.000
Confirmed Triple I	0	(0.0)	0	(0.0)	0.00	1.000
STORCHL-Infection	0	(0.0)	2	(2.2)	0.00	1.000
Streptococcus B Colonization	1	(50.0)	6	(12.0)	6.87	0.253
Bleeding Immediatly Prior to Birth	0	(0.0)	1	(1.1)	0.00	1.000
Partial Premature Placental Abruption	0	(0.0)	4	(4.5)	0.00	1.000
Premature Plazenta Abruption	0	(0.0)	2	(2.2)	0.00	1.000

Table 4: Neonatal Characteristics

	**Recent Betamethasone** median	(range) [n=8]	**Past Betamethasone** median	(range) [n=89]	Δmean	p-value
Birthweight [g]	2020	(1200 - 2720)	2320	(1390 - 3420)	-307.32	0.151
Birthweight z-score	-0.82	(-2.76 - 0.42)	-0.46	(-2.47 - 1.33)	-0.52	0.422
Length at Birth [cm]	44	(29.5 - 50)	46.5	(38 - 51)	-2.89	0.257
Length at Birth z-score	-0.83	(-5.9 - 0.96)	-0.45	(-3.39 - 1.32)	-0.87	0.464
Head Circumference [cm]	30.25	(26.5 - 33)	32	(28 - 36)	-1.61	0.047
Head Circumference z-score	-1.46	(-3.46 - 0.24)	-0.56	(-3.35 - 2.17)	-0.86	0.169
	no.	(%) [n=8]	no.	(%) [n=89]	odds ratio	p-value
Assigned Sex (m:f)	4:4	(50:50)	48:41	(54:46)	0.86	1.000
Small-for-Gestational-Age	3	(37.5)	19	(21.3)	2.19	0.376

Table 5: Neonatal Outcome - Death

	Recent Betamethasone		Past Betamethasone			
	no.	(%) [n=8]	no.	(%) [n=89]	odds ratio	p-value
Stillbirth	0	(0.0)	0	(0.0)	0.00	1.000
Death During First 72h of Life	0	(0.0)	0	(0.0)	0.00	1.000
Death During Neonatal Period (28d)	0	(0.0)	0	(0.0)	0.00	1.000
Composite Outcome: Death	0	(0.0)	0	(0.0)	0.00	1.000

Table 6: Neonatal Outcome - Cardiorespiratory

	**Recent Betamethasone**		**Past Betamethasone**			
	no.	(%) [n=8]	no.	(%) [n=89]	odds ratio	p-value
Supported Transition	4	(50)	27	(30.3)	2.27	0.263
Respiratory Transition Disorder	5	(62.5)	40	(44.9)	2.03	0.466
Intubation	0	(0)	2	(2.2)	0.00	1.000
HighFlow Nasal Cannula	0	(0)	3	(3.4)	0.00	1.000
Continuous Positive Airway Pressure	5	(62.5)	33	(37.1)	2.80	0.256
Inspired Oxygen Fraction >0.3	1	(12.5)	18	(20.2)	0.57	1.000
Any Respiratory Support	5	(62.5)	34	(38.2)	2.67	0.261
CPAP/HFNC >2h	0	(0)	22	(24.7)	0.00	0.192
CPAP/HFNC >12h	0	(0)	16	(18)	0.00	0.346
FiO2 >0.3 longer than 4h	0	(0)	3	(3.4)	0.00	1.000
FiO2 >0.3 longer than 24h	0	(0)	0	(0)	0.00	1.000
Extracorporal Membrane Oxygenation	0	(0)	0	(0)	0.00	1.000
Surfactant	0	(0)	1	(1.1)	0.00	1.000
Apnoe	2	(25)	19	(21.3)	1.23	1.000
Pneumothorax	0	(0)	0	(0)	0.00	1.000
Mekonium Aspiration Syndrome	0	(0)	0	(0)	0.00	1.000
Bronchopulmonary Dysplasia	0	(0)	0	(0)	0.00	1.000
PPHN	0	(0)	0	(0)	0.00	1.000
Persistent Fetal Circulation	0	(0)	0	(0)	0.00	1.000
Arterial Hypotension	0	(0)	4	(4.5)	0.00	1.000
Hemodynamically Relevant PDA	0	(0)	0	(0)	0.00	1.000
PDA: Ibuprofene Therapy	0	(0)	0	(0)	0.00	1.000
Composite Outcome: Kardiorespiratory	0	(0)	24	(27)	0.00	0.194
	median	(range) [n=8]	median	(range) [n=89]	Δmean	p-value
Umbilical Artery pH	7.34	(7.23 - 7.37)	7.32	(7.16 - 7.46)	0.00	0.898
Umbilical Vein pH	7.38	(7.29 - 7.4)	7.38	(7.21 - 7.55)	-0.01	0.908
APGAR min. 1	8	(5 - 9)	9	(4 - 10)	-0.72	0.140
APGAR min. 5	9	(7 - 10)	10	(6 - 10)	-0.23	0.503
APGAR min. 10	10	(9 - 10)	10	(7 - 10)	0.21	0.451
Invasive Ventilation [h]	0	(0 - 0)	0	(0 - 92)	-1.07	0.158
HFNC [h]	0	(0 - 0)	0	(0 - 51)	-0.82	0.083
CPAP [h]	0.11	(0 - 0.2)	0	(0 - 95.5)	-9.08	0.613
Overall Need for Respiratory Support [h]	0.11	(0 - 0.2)	0	(0 - 109.25)	-10.97	0.693
Inspired Oxygen Fraction >0.3 [h]	0	(0 - 0.02)	0	(0 - 22.75)	-0.58	0.375
Treatment With Caffeine [d]	0	(0 - 0)	0	(0 - 15)	-0.98	<0.001
Need for Volume Administration [n]	0	(0 - 0)	0	(0 - 1)	-0.03	0.083
Need for Katecholamine Treatment [d]	0	(0 - 0)	0	(0 - 4)	-0.04	0.320

Table 7: Subgroup Analysis - Neonates With Adverse Respiratory Outcome

	**Recent Betamethasone** no.	(%) [n=5]	**Past Betamethasone** no.	(%) [n=34]	odds ratio	p-value
Supported Transition	4	(80)	27	(79.4)	1.04	1.000
Respiratory Transition Disorder	5	(100)	32	(94.1)	Inf	1.000
Intubation	0	(0)	2	(5.9)	0.00	1.000
HighFlow Nasal Cannula	0	(0)	3	(8.8)	0.00	1.000
Continuous Positive Airway Pressure	5	(100)	33	(97.1)	Inf	1.000
Inspired Oxygen Fraction >0.3	1	(20)	18	(52.9)	0.23	0.342
Any Respiratory Support	5	(100)	34	(100)	0.00	1.000
CPAP/HFNC >2h	0	(0)	22	(64.7)	0.00	0.011
CPAP/HFNC >12h	0	(0)	16	(47.1)	0.00	0.066
FiO2 >0.3 longer than 4h	0	(0)	3	(8.8)	0.00	1.000
FiO2 >0.3 longer than 24h	0	(0)	0	(0)	0.00	1.000
Extracorporal Membrane Oxygenation	0	(0)	0	(0)	0.00	1.000
Surfactant	0	(0)	1	(2.9)	0.00	1.000
Apnoe	2	(40)	14	(41.2)	0.95	1.000
Pneumothorax	0	(0)	0	(0)	0.00	1.000
Mekonium Aspiration Syndrome	0	(0)	0	(0)	0.00	1.000
Bronchopulmonary Dysplasia	0	(0)	0	(0)	0.00	1.000
PPHN	0	(0)	0	(0)	0.00	1.000
Persistent Fetal Circulation	0	(0)	0	(0)	0.00	1.000
Arterial Hypotension	0	(0)	3	(8.8)	0.00	1.000
Hemodynamically Relevant PDA	0	(0)	0	(0)	0.00	1.000
PDA: Ibuprofene Therapy	0	(0)	0	(0)	0.00	1.000
Composite Outcome: Kardiorespiratory	0	(0)	23	(67.6)	0.00	0.008
	median	(range) [n=5]	median	(range) [n=34]	Δmean	p-value
Umbilical Artery pH	7.34	(7.23 - 7.37)	7.32	(7.22 - 7.46)	-0.01	0.895
Umbilical Vein pH	7.36	(7.29 - 7.4)	7.38	(7.27 - 7.55)	-0.03	0.372
APGAR min. 1	7	(5 - 8)	7	(4 - 10)	-0.55	0.429
APGAR min. 5	8	(7 - 9)	8	(6 - 10)	0.11	0.789
APGAR min. 10	10	(9 - 10)	9	(7 - 10)	0.66	0.057
Invasive Ventilation [h]	0	(0 - 0)	0	(0 - 92)	-2.80	0.160
HFNC [h]	0	(0 - 0)	0	(0 - 51)	-2.14	0.083
CPAP [h]	0.17	(0.08 - 0.2)	10.54	(0 - 95.5)	-23.86	<0.001
Overall Need for Respiratory Support [h]	0.17	(0.08 - 0.2)	12.38	(0.07 - 109.25)	-28.80	<0.001
Inspired Oxygen Fraction >0.3 [h]	0	(0 - 0.02)	0.05	(0 - 22.75)	-1.53	0.007
Treatment With Caffeine [d]	0	(0 - 0)	0	(0 - 15)	-1.85	<0.001
Need for Volume Administration [n]	0	(0 - 0)	0	(0 - 1)	-0.06	0.160
Need for Katecholamine Treatment [d]	0	(0 - 0)	0	(0 - 4)	-0.12	0.325

Table 8: Neonatal Outcome - Infection

	**Recent Betamethasone** no.	(%) [n=8]	**Past Betamethasone** no.	(%) [n=89]	odds ratio	p-value
Antibiotic Therapy	1	(12.5)	11	(12.4)	1.01	1.000
Neonatal Infection/Sepsis	0	(0.0)	4	(4.5)	0.00	1.000
Pneumonia	0	(0.0)	0	(0.0)	0.00	1.000
Necrotizing Enterocolitis	0	(0.0)	0	(0.0)	0.00	1.000

Table 9: Neonatal Outcome - Neurologic

	Recent Betamethasone no. (%) [n=8]		Past Betamethasone no. (%) [n=89]		odds ratio	p-value
Asphyxia	0	(0.0)	0	(0.0)	0.00	1.000
Therapeutic Hypothermia	0	(0.0)	0	(0.0)	0.00	1.000
Neurologic Sequelae	0	(0.0)	0	(0.0)	0.00	1.000
Subependymal Hemorrhage	0	(0.0)	2	(2.2)	0.00	1.000
Intraventricular Hemorrhage Grade II	0	(0.0)	0	(0.0)	0.00	1.000
Intraventricular Hemorrhage Grade III	0	(0.0)	0	(0.0)	0.00	1.000
Composite Outcome: Neurologic	0	(0.0)	2	(2.2)	0.00	1.000

Table 10: Neonatal Outcome - Metabolic

	**Recent Betamethasone**		**Past Betamethasone**			
	no.	(%) [n=8]	no.	(%) [n=89]	odds ratio	p-value
Early Feeding (30min From Birth)	5	(71.4)	50	(58.1)	1.79	0.696
Hypoglycemia <2.5 mmol/l	3	(37.5)	14	(15.7)	3.16	0.143
Symptomatic Hypoglycemia OR <1.8 mmol/l	1	(12.5)	3	(3.4)	4.00	0.295
Oral OR i.v. Substitution of Glucose	6	(75)	43	(48.3)	3.17	0.268
Glucose Containing Infusion	6	(75)	43	(48.3)	3.17	0.268
Composite Outcome: Metabolic	6	(75)	45	(50.6)	2.90	0.274
	median	(range) [n=8]	median	(range) [n=89]	Δmean	p-value
Umbilical Vessel Glucose [mmol/l]	3.4	(2.2 - 4.6)	3.8	(1.9 - 6.8)	-0.55	0.161
Lowest Blood-Glucose [mmol/l]	2.95	(1.5 - 4.2)	3.1	(1.3 - 5.7)	-0.21	0.663
Glucose Containing Infusion – Prophylaxis [d]	2	(0 - 3)	0	(0 - 1)	1.21	0.113
Glucose Containing Infusion – Hypoglycemia Therapy [d]	0	(0 - 6)	0	(0 - 1)	1.21	0.248
Glucose Containing Infusion – Other Cause [d]	0	(0 - 0)	0	(0 - 1)	-0.12	0.001
Glucose Containing Infusion – Overall [d]	2.5	(0 - 9)	0	(0 - 2)	2.29	0.039

Table 11: Neonatal Outcome - Icterus

	**Recent Betamethasone** no.	(%) [n=8]	**Past Betamethasone** no.	(%) [n=89]	odds ratio	p-value
Polyglobulia	1	(12.5)	5	(5.6)	2.37	0.412
Hyperbilirubinemia	0	(0)	12	(13.5)	0.00	0.590
	median	(range) [n=8]	median	(range) [n=89]	Δmean	p-value
Highest Bilirubin [µmol/l]	129	(93 - 231)	198.5	(34 - 345)	-43.36	0.079
Photo Therapy [n]	0	(0 - 0)	0	(0 - 7)	-0.24	<0.001
Blood Exchange [n]	0	(0 - 0)	0	(0 - 0)	0.00	NaN

Table 12: Neonatal Outcome - Feeding

	**Recent Betamethasone**		**Past Betamethasone**			
	median	(range) [n=8]	median	(range) [n=89]	Δmean	p-value
Latency to First Feeding [min]	30	(0 - 90)	42	(0 - 375)	-41.81	0.275
Latency to First Own Mothers Milk [d]	2	(0 - 4)	1	(0 - 7)	0.46	0.228
Days With Additional Formula Feds	7.5	(3 - 17)	5	(0 - 26)	0.18	0.527
Days Until Full Enteral Feeding	2.5	(0 - 9)	0	(0 - 12)	1.30	0.175
Gastric Tube Feeding [d]	4.5	(0 - 24)	1.5	(0 - 26)	3.80	0.240
Gastroesophageal Reflux [d]	3	(1 - 11)	2	(0 - 20)	1.49	0.094
	no.	(%) [n=8]	no.	(%) [n=89]	odds ratio	p-value
Ablactation	1	(12.5)	14	(15.7)	0.77	1.000
Additional Formula Feeding at Discharge	4	(57.1)	41	(54.7)	1.10	1.000
Composite Outcome: Feeding	5	(62.5)	55	(61.8)	1.03	1.000

Table 13: Neonatal Outcome - Temperature Regulation

	Recent Betamethasone		Past Betamethasone			
	no.	(%) [n=8]	no.	(%) [n=89]	odds ratio	p-value
Temperature Regulation Disorder	8	(100)	77	(86.5)	Inf	0.590
Composite Outcome: Temperature Regulation	8	(100)	66	(74.2)	Inf	0.192
	median	(range) [n=8]	median	(range) [n=89]	Δmean	p-value
Need for Incubator [d]	0	(0 - 7)	0	(0 - 14)	-0.15	0.864
Need for Warm Bed / Heat Lamp [d]	10	(3 - 17)	6	(0 - 32)	2.81	0.071
Overall Need for External Heat [d]	10	(4 - 22)	8	(0 - 32)	2.88	0.129

Table 14: Neonatal Outcome - Hospital Stay

	Recent Betamethasone no.	(%) [n=8]	Past Betamethasone no.	(%) [n=89]	odds ratio	p-value
Rooming In on the 1st Day of Life	0	(0)	23	(25.8)	0.00	0.192
Neonatal Intensive Care Unit Stay	4	(50)	27	(30.3)	2.27	0.263
Length of Hospital Stay >21 Days	1	(12.5)	17	(19.1)	0.61	1.000
Discharge at Gestational Age >40/0 Weeks	0	(0)	0	(0)	0.00	1.000
Composite Outcome: Hospital Stay	4	(50)	33	(37.1)	1.69	0.476
	median	(range) [n=8]	median	(range) [n=89]	Δmean	p-value
NICU Stay [d]	0.5	(0 - 8)	0	(0 - 23)	0.25	0.400
Hospital Stay [d]	13	(8 - 35)	12	(3 - 38)	3.25	0.197
Gestational Age at Dismission [d]	257	(250 - 274)	255.5	(247 - 280)	1.44	0.919
Weight at Dismission [g]	2157.5	(1860 - 2440)	2400	(1410 - 3165)	-271.2	0.007

Table 15: Neonatal Outcome - Composite Outcomes

	Recent Betamethasone		Past Betamethasone			
	no.	(%) [n=8]	no.	(%) [n=89]	odds ratio	p-value
Composite Outcome: Death	0	(0.0)	0	(0.0)	0.00	1.000
Composite Outcome: Cardiorespiratory	0	(0.0)	24	(27.0)	0.00	0.194
Composite Outcome: Neurologic	0	(0.0)	2	(2.2)	0.00	1.000
Composite Outcome: Metabolic	6	(75.0)	45	(50.6)	2.90	0.274
Composite Outcome: Feeding	5	(62.5)	55	(61.8)	1.03	1.000
Composite Outcome: Temperature Regulation	8	(100.0)	66	(74.2)	Inf	0.192
Composite Outcome: Hospital Stay	4	(50.0)	33	(37.1)	1.69	0.476
Composite Outcome: Combined	8	(100.0)	79	(88.8)	Inf	1.000

Table 16: Sources of BIAS

	Recent Betamethasone		Past Betamethasone			
	no.	(%) [n=8]	no.	(%) [n=89]	odds ratio	p-value
Transfer to Close-To-Home Hospital Prior Discharge	0	(0.0)	1	(1.1)	0.00	1.000
Discharge Against Physician's Advice	0	(0.0)	0	(0.0)	0.00	1.000
Incomplete Documentation	1	(12.5)	3	(3.4)	4.00	0.295
Composite Outcome: BIAS	1	(12.5)	4	(4.5)	2.98	0.356
